# *Shigella* Iron Acquisition Systems and their Regulation

**DOI:** 10.3389/fcimb.2016.00018

**Published:** 2016-02-09

**Authors:** Yahan Wei, Erin R. Murphy

**Affiliations:** ^1^Department of Biological Sciences, Ohio UniversityAthens, OH, USA; ^2^Department of Biomedical Sciences, Heritage College of Osteopathic Medicine, Ohio UniversityAthens, OH, USA

**Keywords:** iron acquisition, *Shigella*, regulation, pathogenicity, virulence

## Abstract

Survival of *Shigella* within the host is strictly dependent on the ability of the pathogen to acquire essential nutrients, such as iron. As an innate immune defense against invading pathogens, the level of bio-available iron within the human host is maintained at exceeding low levels, by sequestration of the element within heme and other host iron-binding compounds. In response to sequestration mediated iron limitation, *Shigella* produce multiple iron-uptake systems that each function to facilitate the utilization of a specific host-associated source of nutrient iron. As a mechanism to balance the essential need for iron and the toxicity of the element when in excess, the production of bacterial iron acquisition systems is tightly regulated by a variety of molecular mechanisms. This review summarizes the current state of knowledge on the iron-uptake systems produced by *Shigella* species, their distribution within the genus, and the molecular mechanisms that regulate their production.

## Introduction

*Shigella* is a genus of Gram negative, facultative anaerobic, pathogenic enterobacteria, composed of four species (*S. boydii, S. sonnei, S. flexneri*, and *S. dysenteriae*), each a causative agent of dysentery in humans. Like all invading pathogens, *Shigella* experiences a wide variety of environmental conditions during transmission and throughout the course of a natural infection. The survival of *Shigella*, and thus its ability to cause disease, is strictly dependent on the ability of the organism to acquire iron from each encountered environment. The strict requirement for iron stems from the fact that this element is an essential co-factor of several enzymes involved in basic biological processes such as DNA replication and respiration. While iron is essential, too much of the element is toxic to a bacterium (Imlay et al., 1988). To balance the necessity and potential toxicity of the element, iron homeostasis must be precisely maintained, a requirement that is facilitated, at least in part, by regulation of bacterial iron-acquisition systems.

As an innate immune defense against invading pathogens, iron within the human body is sequestrated within iron binding compounds and proteins, generating a concentration of bioavailable iron of approximately 10^−24^ M, a concentration that is far below the 10^−7^ M required for the survival of most bacteria (Andrews et al., 2003; Raymond et al., 2003). In response to this iron limitation, pathogenic bacteria have evolved several systems to utilize the various sources of iron present within the infected host. Regulating the production of specific iron acquisition systems in response to environmental conditions allows the bacterium to both efficiently utilize the iron present within a given environment and to avoid iron-mediated toxicity. This review briefly summarizes the current state of knowledge regarding the function and regulation of iron-uptake systems in *Shigella* species.

## Iron-uptake systems in *Shigella*

The currently identified *Shigella* iron-uptake systems can be grouped into three broad categories based on the form of iron that they facilitate the utilization of: (1) systems for the utilization of ferric iron (Fe^3+^), (2) systems for the utilization of heme-bound iron, and (3) systems for the utilization of ferrous iron (Fe^2+^). Each of the four *Shigella* species contains multiple iron uptake systems, however, the combination of iron acquisition systems present vary by species. Each identified *Shigella* iron-uptake system and their distribution among *Shigella* species is presented below and is summarized in Table 1.

**Table 1:** **Summary of the iron-uptake systems in *Shigella* and the regulatory factors**.

**Substrates**	**Iron-uptake systems**	**Effects of identified regulatory factors**	**Distribution among *Shigella* species**
Fe^3+^	Ent/Fep	Fe (↓)	S. sonnei, S. dysenteriae, S. boydii^*^, S. flexneri^*^
	Iro	Fe (↓)	*S. dysenteriae*
	Iuc/Iut	Fe (↓), O_2_ (↑), RhyB (↑) in *E. coli*	*S. sonnei, S. boydii, S. flexneri*
	Fec	Fe (↓), ECF (↑)	*S. sonnei, S. flexneri*
	Fhu	Fe (↓), O_2_ (↑)	*S. sonnei, S. dysenteriae, S. boydii, S. flexneri*
Heme	Shu	Fe (↓), High temperature (↑)	*S. dysenteriae, S. sonnei*
Fe^2+^	Feo	Fe (↓), O_2_ (↓)	*S. sonnei, S. dysenteriae, S. boydii, S. flexneri*
	Sit	Fe (↓), O_2_ (↑)	*S. sonnei, S. dysenteriae, S. boydii, S. flexneri*
	Efe	Fe (↓), low pH (↑)	*S. sonnei*

* *The biosynthetic system of enterobactin in S. boydii and S. flexneri is inactivated by the presence of a frameshift, a premature stop codon, and/or an insertion within the coding genes; as a consequence, no detectable enterobactin is produced*.

### Ferric iron utilization systems

**Figure 1:**
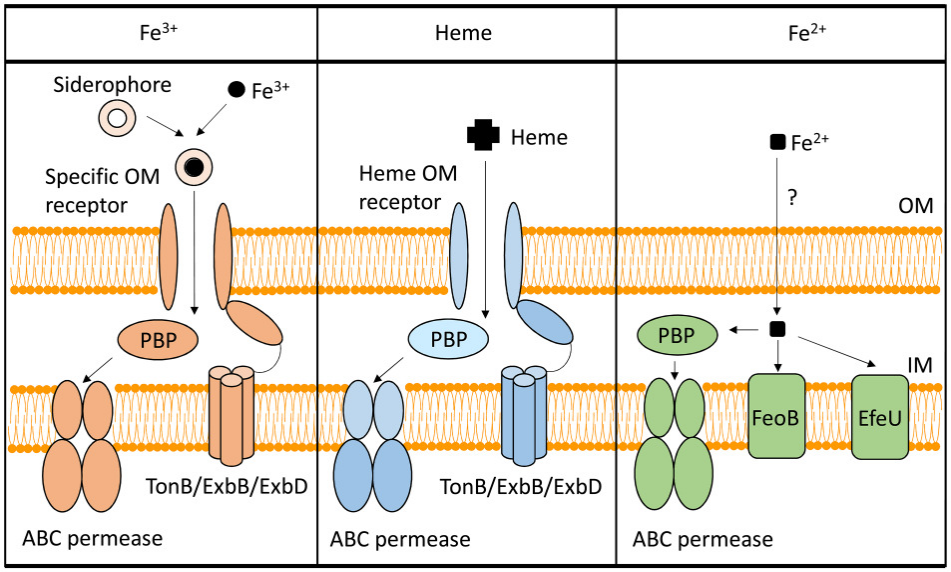
**Iron-uptake systems in *Shigella***. This figure is a schematic of the *Shigella* iron-uptake systems categorized into three broad groups based on the form of iron or iron-containing compounds being utilized. These three groups are (1) systems for the utilization of ferric iron (Fe^3+^), (2) systems for the utilization of heme (Heme), and (3) systems for the uptake of ferrous iron (Fe^2+^). Within the ferrous iron uptake systems, the PBP represents SitA, while the ABC permease represents SitBCD. “PBP” stands for periplasmic binding protein.

To utilize Fe^3+^, *Shigella* have evolved several systems to synthesize, secrete, and uptake siderophores, a group of compounds with high affinity for iron that functionally compete for iron bound by iron-sequestrating factors within the infected host (Carrano and Raymond, 1979; Hider and Kong, 2010). In *Shigella*, the combination of siderophores that are synthesized and utilized varies by species, but the uptake process is generally conserved (Figure 1). Specifically, transport of each Fe^3+^-siderophore into the bacterium is initiated by binding of the complex to a specific outer-membrane receptor. Once bound by its receptor, the complex is transported across the outer-membrane, passed to a periplasmic binding protein (PBP), and finally transported across the inner-membrane by the activity of an ABC permease complex. Compared to that of the outer-membrane receptors, PBPs have lower substrate specificity, thus one PBP can facilitate the transportation of multiple siderophores with similar chemical structures (Miethke and Marahiel, 2007). Transport of any cargo across a membrane requires energy. Anchored within the inner-membrane, the TonB/ExbB/ExbD complex transduces energy generated by the proton gradient to a given iron-binding outer-membrane receptor to provide the energy required to transport the associated cargo across the outer-membrane (Larsen et al., 1997). ATP hydrolysis generates the energy needed for the subsequent transport of the cargo across the inner-membrane. Once inside the bacterial cell, iron is either released from the siderophore for utilization, or bound within storage proteins for future use. Each identified *Shigella* siderophore and its transport system is discussed below.

#### Enterobactin

Discovered in 1970, enterobactin is the siderophore with the highest known affinity for Fe^3+^ (~10^49^) (O'Brien et al., 1970; Pollack and Neilands, 1970; Loomis and Raymond, 1991). Genes involved in the synthesis, secretion, and uptake of enterobactin are encoded in a single locus, with *ent* genes encoding factors involved in the synthesis/secretion of the siderophore and *fep* genes encoding factors composing the uptake system (Laird et al., 1980). Specifically, *fepA* encodes a TonB-dependent outer-membrane receptor, *fepB* encodes a PBP, and *fepCDG* encodes proteins constituting the ABC permease complex (Ozenberger et al., 1987). All four *Shigella* species contain the *ent/fep* locus, however some of these genes are inactivated in *S. boydii* and in some strains of *S. flexneri*, due to the presence of a frameshift, a premature stop codon, and/or an insertion mutation (Payne, 1980; Payne et al., 1983).

Due to its high iron-binding affinity, enterobactin can chelate the element from various host iron-binding factors (Carrano and Raymond, 1979). For example, a recent study demonstrates that enterobactin can overcome the sequestration of iron by ATP within the intracellular environment, and as such would promote survival of the pathogen within a macrophage (Tatano et al., 2015). When present in the extracellular environment however, bacteria often induce host cells to produce and secrete lipocalin-2, a protein that specifically binds enterobactin, thus preventing the bacterium from utilizing the iron bound within it (Flo et al., 2004).

#### Salmochelin

Due to their different chemical structures, some enterobactin derivatives are not recognized by lipocalin-2 and, therefore not surprisingly, have been found to contribute to virulence. One such enterobactin derivative is salmochelin, a siderophore produced by *S. dysenteriae* (Fischbach et al., 2006; Wyckoff et al., 2009). The *iro* locus contains genes encoding enzymes that transform enterobactin into salmochelin, the machinery required to secrete salmochelin, and a salmochelin specific TonB-dependent outer-membrane receptor (Hantke et al., 2003). Additional factors involved in salmochelin utilization, including the PBP and the ABC transporter, are the same as those used for the utilization of enterobactin (Müller et al., 2009).

#### Aerobactin

Some strains of *S. flexneri, S. boydii*, and *S. sonnei* produce aerobactin, a siderophore that has a different chemical structure from that of enterobactin and as a result, can also escape the sequestration by host protein lipocalin-2 (Lawlor and Payne, 1984; Flo et al., 2004). Aerobactin has been shown to promote the virulence of uropathogenic *Escherichia coli*, and to facilitate extracellular growth of *Shigella* (de Lorenzo and Martinez, 1988; Torres et al., 2001). *iucABCD* encodes the enzymes required for the synthesis of aerobactin and is found within a single locus along with *iutA*, a gene encoding the aerobactin-specific TonB-dependent outer-membrane receptor (Carbonetti and Williams, 1984). The remaining factors required for the utilization of aerobactin are encoded within the *fhu* locus, and are also utilized for the transportation of ferrichrome (see below; Köster and Braun, 1990).

#### Xenosiderophores

Like many other bacterial species, *Shigella* can utilize xenosiderophores, siderophores produced by other microorganisms (Payne, 1980). For example, ferrichrome, a fungal siderophore with a chemical structure similar to that of aerobactin is utilized by *Shigella* species. The utilization of ferrichrome is mediated by factors composing the Fhu system including FhuA, a ferrichrome-specific TonB-dependent outer-membrane receptor (Köster and Braun, 1990; Miethke and Marahiel, 2007).

#### Ferric-dicitrate

Ferric iron can bind with citrate to form ferric-dicitrate. *S. sonnei* and at least one strain of *S. flexneri* can utilize ferric-dicitrate as a source of nutrient iron (Luck et al., 2001; Wyckoff et al., 2009). The utilization of ferric-dicitrate bound iron is mediated by factors encoded within the *fec* locus, including an outer-membrane receptor (FecA), a PBP (FecB), and an ABC transport complex (FecCDE) (Braun and Mahren, 2005).

### Heme utilization system

The *Shigella* heme-uptake (Shu) system was first identified in *S. dysenteriae* and is predicted to be present in some strains of *S. sonnei* (Wyckoff et al., 1998). Inactivation of *shuA*, a gene encoding the outer-membrane heme receptor, eliminates the ability of *S. dysenteriae* to utilize heme as a sole source of nutrient iron, suggesting that the Shu system is the only functional heme-utilization system in this species (Mills and Payne, 1997). In uropathogenic *E. coli*, inactivation of the orthologous gene (*chuA*) results in attenuation of virulence, a finding that directly identifies the heme receptor as a virulence factor in this closely related species (Torres et al., 2001). Additional components of the Shu system include a PBP (ShuT) and an inner-membrane ABC permease complex (ShuUV) (Wyckoff et al., 1998; Eakanunkul et al., 2005; Burkhard and Wilks, 2008). The process of heme-uptake is similar to that detailed above for the uptake of siderophores (Figure 1). Interestingly, the fact that *Shigella* species which lack the *shu* genes are able to utilize heme-bound iron suggests the existence of a yet unidentified heme-utilization system(s) (Payne et al., 2006).

### Ferrous iron utilization systems

Under anaerobic and/or acidic conditions, Fe^2+^ is the dominant form of the element. Three Fe^2+^-uptake systems have been identified in *Shigella*: the Feo system, the Sit system, and the Efe system (Kammler et al., 1993; Zhou et al., 1999; Jin et al., 2002; Große et al., 2006; Figure 1).

#### Feo system

Despite being the first Fe^2+^ utilization system identified in *Shigella*, details of the molecular mechanism(s) underlying the activity of the Feo system remain largely unknown (Kammler et al., 1993). Only three components of the Feo system have been identified to date: FeoA, FeoB, and FeoC. FeoB is an inner-membrane transporter with GTPase activity and a structure similar to that of a eukaryotic G protein (Marlovits et al., 2002). FeoC contains an oxygen-responsive [4Fe-4S] cluster and functions to promote proteolysis of FeoB in the presence of oxygen (Hsueh et al., 2013; Kim et al., 2015). While known to be required for the transport of Fe^2+^ by FeoB, the exact function of FeoA remains to be determined (Kim et al., 2012; Lau et al., 2013).

#### Sit system

The *sit* locus contains four genes: *sitA* encoding the PBP and *sitBCD* encoding components of the ABC permease complex (Zhou et al., 1999; Fisher et al., 2009). Interestingly, no outer-membrane receptor has been identified to date, leading to the hypothesis that Fe^2+^ is transported through the outer-membrane via non-specific porins and/or ion channels (Andrews et al., 2003). Interestingly, studies in *Salmonella* indicate that SitA has a higher affinity for manganese than for Fe^2+^, suggesting that the primary function of the Sit system might be to transport manganese (Kehres et al., 2002). It has been demonstrated however, that *S. flexneri* is able to use the Sit system as the sole iron-uptake system to survive and form plaques in a monolayer of eukaryotic cells, suggesting not only that the system can function to uptake iron but also that the Sit system has a direct role in pathogenesis (Runyen-Janecky et al., 2003). It remains a formal possibility that the *Shigella* Sit system functions to transport both manganese and iron. Interestingly, the *E. coli* orthologous of the *Shigella* manganese transporter MntH and zinc transporter YgiE (MntH and ZupT, respectively) have been shown to transport Fe^2+^ in addition to their specific substrates, findings that support the hypothesis that additional Fe^2+^ transport systems may exist in *Shigella* species (Kehres et al., 2000; Grass et al., 2005).

#### Efe system

Originally characterized in *E. coli*, genetic analysis demonstrates that the EfeUOB system, formally called YcdNOB, is encoded by genes present on a tricistronic transcript in at least one strain of *S. sonnei* (Große et al., 2006; Cao et al., 2007; Payne and Alexandra, 2010). *efeU* encodes the inner membrane permease and is homologous to the yeast Fe^2+^ transporter Ftr1p (Große et al., 2006). EfeO and EfeB are both periplasmic proteins necessary for Fe^2+^ transport, however their specific functions remain unknown.

## Regulation of iron-uptakes systems

To ensure that a given iron acquisition system is optimally produced under the specific environmental condition(s) in which its function will be most advantageous to the pathogen, production of *Shigella* iron-uptake systems is tightly controlled by multiple regulatory factors via distinct molecular mechanisms (Table 1). The major regulatory mechanisms governing the production of *Shigella* iron acquisition systems are detailed below.

### Regulation by extracytoplasmic function (ECF) sigma factors

The *Shigella* Fec system, utilized for the uptake of ferric-dicitrate, is regulated by the ECF sigma factor FecI (Lonetto et al., 1994; Braun and Mahren, 2005). Upon binding of ferric-dicitrate to FecA, the TonB-dependent outer-membrane receptor undergoes a conformational change resulting in the interaction of its N-terminal domain with the C-terminal domain of the trans-membrane anti-sigma factor FecR. The interaction of FecA with FecR results in the release of the alternative sigma factor FecI that, in turn, directs RNA polymerases to the alternative promoter region of the *fecABCDE* operon (Braun and Mahren, 2005). Such regulation results directly in increased production of the Fec system when *Shigella* is within an environment containing ferric citrate.

### Regulation by Fur

Availability of iron influences the production of several *Shigella* iron-acquisition systems via Fur, an iron-responsive transcriptional regulator (Fleming et al., 1983; de Lorenzo et al., 1987; Wyckoff et al., 1998; Payne et al., 2006). The interaction of Fur with intracellular iron induces a conformational change in the protein that enables it to dimerize and bind DNA in a sequence specific manner (Troxell and Hassan, 2013). Iron-dependent binding of Fur at or near the promoter region of a target gene most often inhibits transcription by physically blocking binding of RNA polymerase (Troxell and Hassan, 2013).

### Regulation by RhyB

RyhB, a Fur-repressed regulatory small RNA first identified in *E. coli*, plays an important role in achieving iron homeostasis in several bacterial species, including *Shigella*. RyhB functions to modulate the stability of specific target transcripts in response to iron availability within the environment (Massé and Gottesman, 2002). Specifically, under iron-poor conditions, Fur-mediated repression of RhyB is relieved, and once produced, the small RNA functions to repress the production of several factors including iron-containing enzymes, such as SodB and iron-storage proteins, such as ferritin, in both *E. coli* and *Shigella* (Massé and Gottesman, 2002; Murphy and Payne, 2007). In *E. coli*, RhyB also regulates the production of aerobactin, however the details of this regulatory mechanism, as well as the role that such a mechanism may play in controlling *Shigella* gene expression remains to be revealed (Porcheron et al., 2014).

### Oxygen-dependent regulation

Within a given environment, the amount of oxygen influences the relative abundance of Fe^3+^ and Fe^2+^; thus it is reasonable that bacterial iron-uptake systems are also regulated in response to environmental oxygen levels. Two regulatory systems with over-lapping, yet non-identical regulons control the production of *Shigella* bacterial iron acquisition systems in response to oxygen status: the two component system ArcAB, and the DNA-binding regulator Fnr (Carpenter and Payne, 2014).

#### ArcAB system

Within the ArcAB two-component system, ArcB is a membrane-anchored kinase and ArcA is a DNA-binding response regulator (Carpenter and Payne, 2014). Following auto-phosphorylation of ArcB, a process that occurs only under anaerobic conditions, the activated kinase phosphorylates ArcA. Once phosphorylated, ArcA functions to directly alter the expression of specific target genes (Carpenter and Payne, 2014). Under aerobic conditions, the auto-phosphorylation of ArcB is inhibited, ultimately resulting in the lack of ArcA activation and thus altered expression of ArcA-regulated genes. As a global regulator, ArcA not only modulates the production of factors involved in iron-uptake, but also that of the iron-responsive regulator Fur (Boulette and Payne, 2007).

#### Fnr

As a DNA-binding protein, the regulatory mechanism underlying the activity of Fnr is similar to that underlying the activity of Fur. Unlike Fur however, the conformational change controlling the regulatory activity of Fnr is determined directly by the oxidative status of its [4Fe-4S] cluster, and thus indirectly by the oxygen status of the environment (Carpenter and Payne, 2014). With increased levels of intracellular oxygen, the [4Fe-4S] cluster within Fnr is oxidized into [2Fe-2S], a transition that results into the loss of DNA-binding ability, and thus regulatory activity, of the protein.

### Temperature-dependent regulation

A change in environmental temperature to that encountered within the human body (37°C) is an important signal that can indicate a transition from the non-host environment to the human host. As such, temperature influences the expression of many *Shigella* virulence-associated genes, including that of *shuA* (Tobe et al., 1991; Kouse et al., 2013). Production of ShuA, an outer-membrane heme receptor, is subject to temperature-dependent post-transcriptional regulation via the activity of an RNA thermometer located within the 5′ untranslated region of *shuA* (Kouse et al., 2013). RNA thermometers regulate translation of the gene in which they are housed by the formation of an inhibitory structure that physically occludes the ribosomal binding site at relatively low temperatures. At relatively high temperatures, such as that encountered within the human host, the inhibitory structure within an RNA thermometer is destabilized, the ribosomal binding site is exposed and translation proceeds. While ShuA is currently the only *Shigella* iron acquisition factor known to be regulated by the activity of an RNA thermometer, the full impact of this temperature-dependent regulatory mechanism on iron utilization by these pathogens remains to be determined.

## Future prospective

Given the importance of iron homeostasis to survival, the mechanisms by which bacteria acquire iron from the environment and the regulatory mechanisms controlling the production of bacterial iron acquisition systems have long been the subject of active investigation. As highlighted above, the importance of several iron utilization systems to virulence has been proven in other pathogenic bacteria, however, exactly how these factors, and their associated regulation, contribute to *Shigella* virulence remains to be fully elucidated. Moreover, whether the presence of a specific combination of iron uptake systems in a given *Shigella* species contributes to differences in survival within host or non-host environments, differences in geographic distribution and/or differences in pathogenesis would be a potentially revealing analysis, but one that can only be completed after all the iron acquisition systems in these pathogenic organisms are recognized. Finally, future investigations to identify additional *Shigella* iron acquisition systems and to understand the role of these systems in *Shigella* virulence could lead to the development of novel therapeutics designed to disrupt iron acquisition, and by doing so eliminate or reduce the ability of these bacterial pathogens to cause human disease.

## Author contributions

Wrote the article: YW and EM. Edited article: YW and EM. Generated figure: YW. Generated table: YW.

### Conflict of interest statement

The authors declare that the research was conducted in the absence of any commercial or financial relationships that could be construed as a potential conflict of interest.
